# Bridging the Gap in Malaria Parasite Resistance, Current Interventions, and the Way Forward from in Silico Perspective: A Review

**DOI:** 10.3390/molecules27227915

**Published:** 2022-11-16

**Authors:** Ransford Oduro Kumi, Belinda Oti, Nader E. Abo-Dya, Mohamed Issa Alahmdi, Mahmoud E. S. Soliman

**Affiliations:** 1 Molecular Bio-Computation and Drug Design Laboratory, School of Health Sciences, Westville Campus, University of KwaZulu-Natal, Durban 4001, South Africa; 2 Department of Industrial and Health Sciences, Faculty of Applied Sciences, Takoradi Technical University, Takoradi 256, Ghana; 3 Department of Pharmaceutical Chemistry, Faculty of Pharmacy, University of Tabuk, Tabuk 71491, Saudi Arabia; 4 Department of Pharmaceutical Organic Chemistry, Faculty of Pharmacy, Zagazig University, Zagazig 44519, Egypt; 5 Department of Chemistry, Faculty of Science, University of Tabuk, Tabuk 71491, Saudi Arabia

**Keywords:** malaria, plasmodium falciparum, plasmepsin, parasite resistance, computer-aided drug design (CADD)

## Abstract

The past decade has seen most antimalarial drugs lose their clinical potency stemming from parasite resistance. Despite immense efforts by researchers to mitigate this global scourge, a breakthrough is yet to be achieved, as most current malaria chemotherapies suffer the same fate. Though the etiology of parasite resistance is not well understood, the parasite’s complex life has been implicated. A drug-combination therapy with artemisinin as the central drug, artemisinin-based combination therapy (ACT), is currently the preferred malaria chemotherapy in most endemic zones. The emerging concern of parasite resistance to artemisinin, however, has compromised this treatment paradigm. Membrane-bound Ca^2+^-transporting ATPase and endocytosis pathway protein, Kelch13, among others, are identified as drivers in plasmodium parasite resistance to artemisinin. To mitigate parasite resistance to current chemotherapy, computer-aided drug design (CADD) techniques have been employed in the discovery of novel drug targets and the development of small molecule inhibitors to provide an intriguing alternative for malaria treatment. The evolution of plasmepsins, a class of aspartyl acid proteases, has gained tremendous attention in drug discovery, especially the non-food vacuole. They are expressed at multi-stage of the parasite’s life cycle and involve in hepatocytes’ egress, invasion, and dissemination of the parasite within the human host, further highlighting their essentiality. In silico exploration of non-food vacuole plasmepsin, PMIX and PMX unearthed the dual enzymatic inhibitory mechanism of the WM382 and 49c, novel plasmepsin inhibitors presently spearheading the search for potent antimalarial. These inhibitors impose structural compactness on the protease, distorting the characteristic twist motion. Pharmacophore modeling and structure activity of these compounds led to the generation of hits with better affinity and inhibitory prowess towards PMIX and PMX. Despite these headways, the major obstacle in targeting PM is the structural homogeneity among its members and to human Cathepsin D. The incorporation of CADD techniques described in the study at early stages of drug discovery could help in selective inhibition to augment malaria chemotherapy.

## 1. Introduction

Plasmodium (P) parasites, which are spread by the bite of female anophele mosquitoes, are responsible for the crippling disease malaria. At least 3.2 billion of the world’s population stand at risk of contracting malaria, and an estimated 350–500 million clinical malaria cases occur annually, with the majority of the incidents emanating from the sub-Sahara regions [1]. In South Africa, malaria cases and transmission follow a pattern that mostly starts and peaks in October and January, respectively (NICB, 2021).

Currently, *P. falciparum, P. ovale, P. malariae, P. knowlesi*, and *P. vivax* are the five known Plasmodium species to infect humans [2]. Among these species, *P. falciparum* is the most common in sub-Saharan Africa and has been implicated in serious life-threatening malaria conditions and thus tagged as the most lethal [3]. Despite the global awareness and mortality stemming from malaria, the only approved WHO vaccine Mosquirix is scarce even in endemic regions [4].

Herbs and herbal extracts are frequently used in various parts of the world to manage and treat malaria because they are affordable, easily accessible, and effective [5,6]. Curcumin, an active compound of turmeric, has been known to possess antimalarial effects against various *Plasmodium* species. However, the use of herbs in phytotherapy is often poorly standardized and controlled. Moreover, their active substance content may vary according to plant genetics as well as climatic factors. As such, most malaria control strategies today depend on safe and effective drugs, as they have done for decades [7]. The majority of the antimalarial drugs are schizonticidal and thus target the asexual erythrocytic stage of the plasmodium parasite. Other antimalarial drugs aimed at the dormant stage of the parasite in the liver, as well as the sexual erythrocytic forms of the parasite, are in use. Inhibiting the sexual erythrocytic form of the parasite in the bloodstream further prevents the uptake and transmission of malarial by Anopheles mosquitoes.

**Figure 1:**
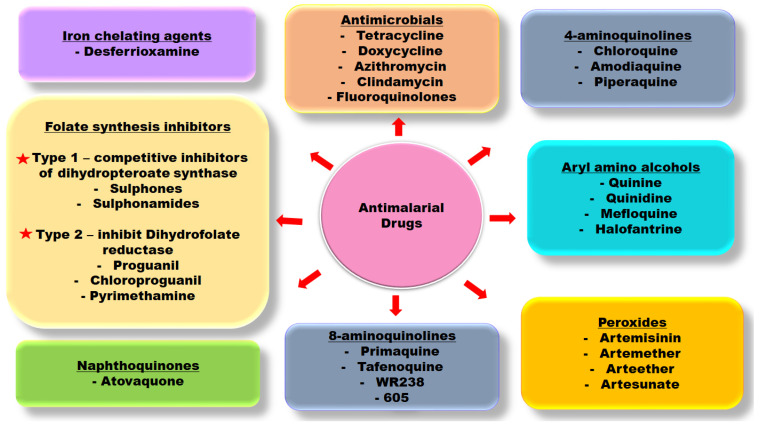
Class of antimalarial drugs in clinical usage.

Artemisinin-based combination therapy (ACT), a drug combination therapy with artemisinin as the central drug, has, over the years, been at the forefront of the treatment of uncomplicated malaria [8]. In the early 90s, the original artesunate combination was with mefloquine in areas of multidrug resistance; however, derivatives of artemisinin generated from structure–activity relationship study are now being evaluated in many other endemic areas in combinations with other antimalarials such as lumefantrine, piperaquine, amodiaquine and sulfadoxine-pyrimethamine [3]. Despite the synergic effect of the combinational therapy, there are growing concerns about drug resistance associated with the central drug, artemisinin, thereby compromising its combinations for malaria treatment [8]. Several classes of antimalarial drugs have been developed and are in clinical usage ranging from the aryl amino alcohol compounds, antifolate compounds, and artemisinins, as represented in Figure 1. The mode of action of selected antimalarial drugs is as well outlined in Table 1.

### 1.1. Parasite Resistance to Antimalarial Drugs; Persistent Concern

**Figure 2:**
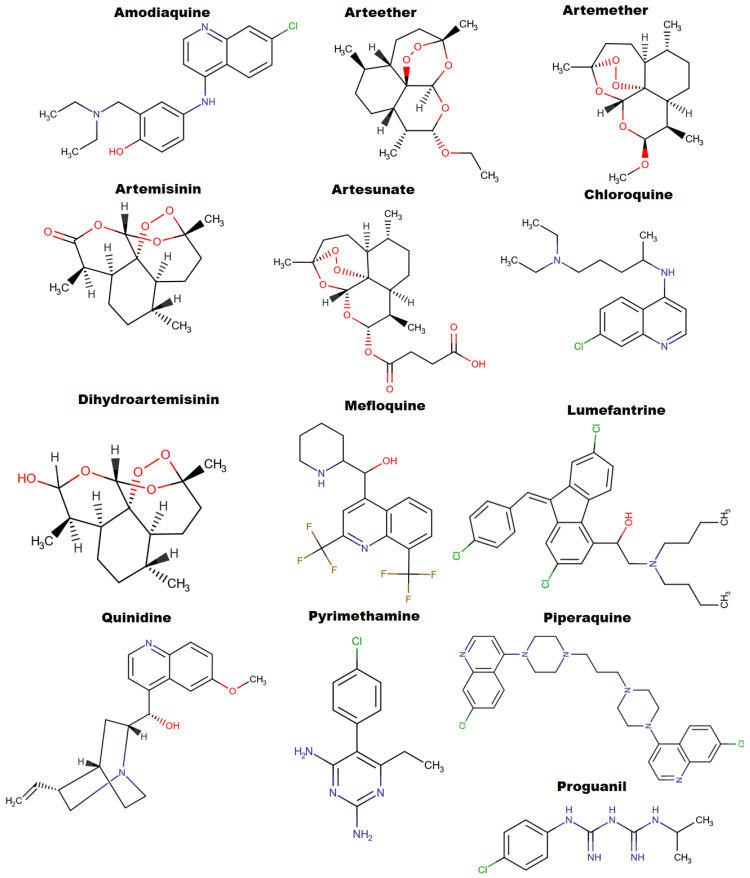
Chemical structural representation of frontline antimalaria drugs in clinical usage.

Antimalarial resistance poses a major obstruction to malaria chemotherapy in sub-Saharan Africa [9] largely because *Plasmodium falciparum* is prone to drug resistance [10]. Resistance is the parasite’s intrinsic proficiency to survive and/or multiply despite the proper administration and absorption of an antimalarial medicine in the recommended dose [11]. The parasite’s broad antigenicity and genetically different stages, in addition to its intricate life cycle, including a mosquito vector and a human host, might be a hindrance to malarial control measures and ultimately increase resistance to chemotherapy. Surveying the spread of resistance is challenging since the mechanism underlying artemisinin resistance is poorly understood, and there are no good genetic markers to track artemisinin resistance [10]. The first reports of chloroquine resistance to Pf in Africa were made in the late 1970s, and the resistance of the malaria strains was linked to a parasite protein called Pf chloroquine resistance transporter (PfCRT), the mutated form of which can reduce chloroquine accumulation in the digestive vacuole of the pathogen [12]. A summary of transporting proteins and other enzymes implicated in malaria parasite resistance to antimalarial drugs is detailed in Table 2. *Plasmodium vivax* is known to put a significant burden on the malaria-endemic world with high morbidity and mortality due to its tendency to cause recurrent infections. Weeks after the initial attack, the dormant liver stages called hypnozoites trigger a relapse of the infection. Recurring infections, which are the primary cause of vivax sickness, can happen as frequently as every three weeks. Chloroquine, the first-line treatment for *P. vivax* malaria in most endemic countries, faces similar setbacks of resistance in different parts of the world [13]. The transmembrane protein P-glycoprotein homologous 1 (Pgh1) is encoded by the *P. falciparum* multidrug resistance gene (pfmdr), which also regulates the resistance phenotype in some PfCRT mutant parasites [14]. Folate pathway enzyme Dihydrofolate reductase (Dhps) mutations exhibited a significant impact on treatment failure in children with high parasitemia. Triple point polymorphism *dhps*-437 and *dhps*-540 recorded a risk of treatment failure of 37% in children with a parasite load of fewer than 45,000 parasites/μL [15]. Chemical structures of frontline antimalaria drugs are shown in Figure 2.

In the quest to overcome the global challenge of parasite resistance, researchers have geared prospective studies toward the investigation of novel drug targets whose inhibition is catastrophic to the parasite. Among these targets are plasmepsins, a family of aspartic acid proteases responsible for hemoglobin degradation, hepatocellular egress, and dissemination of the infection within the host. The role of plasmepsin in malaria treatment is further expatiated in Section 4.

### 1.2. Binding of Mainstay Antimalarial Drugs Reveals Significant Interaction with Glutamic Acid Residue (Glu) in the Catalytic Domain

**Figure 3:**
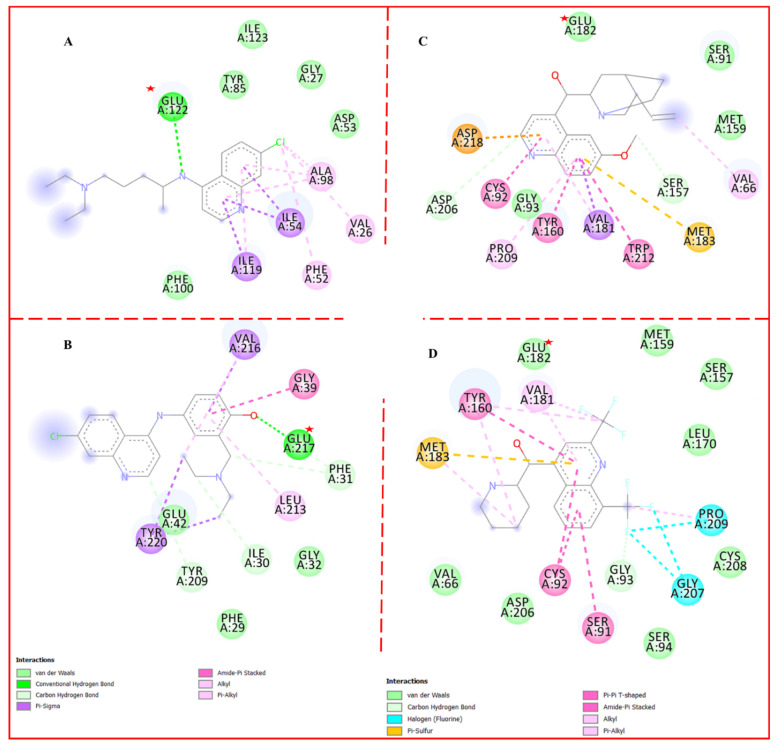
Ligand–receptor interaction profile of mainstay antimalarial drugs highlighting their interaction with Glu. (**A**) Chloroquine in the cofactor binding site of *P. falciparum* lactate dehydrogenase PDB code: 1CET, (**B**) Amodiaquine in complex with Phosphoethanolamine Methyltransferase from *P. falciparum* PDB code 4FGZ: (**C**) *P. falciparum* purine nucleoside phosphorylase in complex with quinine PDB code: 5ZNC and (**D**) *P. falciparum* purine nucleoside phosphorylase in complex with mefloquine PDB code: 5ZNI. Indicates the position of glutamic acid (Glu).

This chapter elucidates the interaction of quinoline front-line antimalarial drugs in the active pockets of respective target proteins of *P. falciparum*. It is, however, intriguing to know that Chloroquine, Amodiaquine, Quinine, and Mefloquine interact significantly with Glutamic acid (Glu) in securing overall stability within the binding pocket. The drugs form a mesh of conventional hydrogen bonds and hydrostatic interaction with Glu, as shown in Figure 3. These interactions emphasize the importance of Glu in the enzyme inhibitory prowess of this class of antimalarials, evoking the thought of point mutation in Glu in parasite resistance. Mutation study in different strains of the plasmodium parasite is imminent in the pursuit of unraveling potent antimalarial drugs. Incorporating fragments or compounds with a higher affinity for Glutamic acid via structure–activity relationship (SAR) study and ligand-based virtual screening (LBVS) in the drug discovery and development process could as well curb parasite resistance. Computer-aided drug design (CADD) techniques in the drug discovery process are described in the later part of the study.

## 2. Computer-Aided Drug Design Approaches to Mitigate the Global Burden of Parasite Resistance

The hunt for innovative pharmacological targets offering a more alluring path toward the effective treatment and relief of malaria has been prompted by the enormous occurrence of malaria and the rising emergence of parasite resistance to conventional therapies [16]. These targets are mostly enzymes that play a significant role in the very survival of the parasite. Inhibiting these proteins thereof is detrimental to the parasite and relief the host of the infection. Identification and development of novel drugs for clinical usage go through several years, and it is cost intensive; moreover, several drug candidates fail at the final stages of clinical trials. The few that progress into clinical usage sometimes face challenges of parasite resistance, thereby hindering their clinical application. The introduction of Computer-Aided Drug Design (CADD) techniques in the drug discovery process have proven vital through the numerous advances over the past decades. CADD techniques not only save cost and fast-track the drug discovery and design process but also provide atomistic and molecular insight into the ligand–receptor interactions. CADD techniques such as molecular dynamic (MD) simulation mimic the interactions of a therapeutic compound and receptor in a living system via dynamic simulation. Additionally, CADD approaches are implemented in predicting the drug-likeness and toxicology of drug candidates, screening of database for potential drug candidates, and predicting of binding mode and affinity of ligands, as well as molecular simulation of ligand–receptor complexes. [17]. This chapter details computational techniques explored in the drug discovery and development process of small molecule inhibitors.

### 2.1. Homology Modeling of Novel Malaria Drug Targets

**Figure 4:**
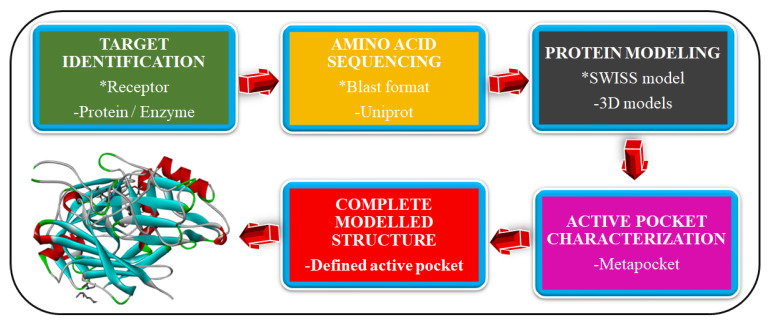
In-house stages employed in homology modeling of target proteins with defined binding pockets.

Ligand–receptor complex is essential to envisage the interaction of drug candidates within the catalytic domain of the receptor. These interactions stem from the formation of bonds, most significantly conventional hydrogen bonds and hydrophobic interactions. In drug discovery, drugs are designed complementary to the active pockets. Computation analysis of the ligand–receptor complex encompasses the use of the crystallized structure of the receptor and the ligand. Early studies on the non-food vacuole plasmepsins were centered on homology modeling as the crystalized structures were unavailable at the protein data bank [18,19]. Homology modeling is a multi-step operation that involves the building of crystalized structures of target proteins from their amino acid sequence. A study by Geraldine et al. (2020) employed homology modeling in building the 3D structures of *P. falciparum* PMIX and PMX against human cathepsin D (4OBZ). The outline of homology modeling techniques is represented in Figure 4. In our previous study by Kumi et al. [20], an online tool UniProt was used to predict the amino acid sequence of the target, PMIX, while the catalytic domain of the protein was mapped using the metapocket tool.

### 2.2. Structure–Activity Relationship (SAR)

Structure–Activity Relationship (SAR) is an online technique intended to find relationships between structural-related properties, mostly between chemical structure and biological activities. The pharmacophoric moiety of the therapeutic compound responsible for eliciting biological effects in the target protein is highlighted, serving as a template to search for drug-like compounds with similar structures. This permits adjustment of the effect or the potency of the bioactive compound by altering its chemical structure. This technique was employed in our previous study to identify compounds with the 2,4-diaminopyrimidine rings in the quest to develop inhibitors for the treatment of Alzheimer’s disease [21]. Similarly, a comprehensive structure–activity relationship study using CoMFA has been explored where artemisitene was used as the transitional compound. Analogs of artemisinin were studied for their antimalarial activity; novel C-9-modified artemisinin analogs showed significant antimalarial activity. Furthermore, the antileishmanial activity of more than 70 artemisinin derivatives against Leishmania donovani promastigotes was as well investigated [22]. Their study recommended the possibility of developing artemisinin analogs as potential drug candidates against both malaria and leishmaniases.

To forecast new in vitro antimalarial medicines, a quantitative structure–activity relationship analysis employing a library of 395 compounds previously evaluated against chloroquine-susceptible strains of the blood stages of *P. falciparum* was carried out [23], the study concluded that monensin, nigericin, vincristine have potent antimalarial prowess with an IC_50_ of 0.35μM, 0.4 μM, and 2.0 μM, respectively, thus demonstrating the essentiality of this approach in the identification of lead compounds against *P. falciparum*. Additionally, azithromycin derivatives from SAR showed a significant enhancement in vitro antimalarial activity against *P. falciparum*; the aromatic moiety on the 9a-position of the sugar-containing macrocyclic ring plays an important role [24].

### 2.3. Visual Screening for Antimalarial Compounds

Virtual screening (VS) is a computational technique engaged in probing libraries of small molecules to identify therapeutic compounds with the tendency to influence drug targets. vs. plays a central role in the early stage of drug discovery and development, one of the productive and cost-effective technology in the search for novel lead compounds [25]. Comparative to high-throughput screening (HTS), vs. searches a large database of chemicals with high accuracy and achieves drug-target relevance. vs. investigations are based on the structure of the target protein or the ligand.

### 2.4. Structure-Based Virtual Screening (SBVS)

Structure-based virtual screening (SBVS) is a computational method performed in the early-stage drug discovery process to search libraries of chemical compounds for novel bioactive molecules against a known drug target. SBVS employs a 3D structure of a target protein of interest which is determined experimentally or through homology modeling, as discussed earlier. The active pocket of the target protein is docked by small molecule inhibitors from the database and is ranked based on their predicted binding affinity or complementarity to the binding site. The outcome of SBVS could be hundreds of compounds, but mostly only a few top-ranked drug-like compounds are selected for further experimental assays. This approach is essential in the identification of hits.

### 2.5. Ligand-Based Virtual Screening (LBVS)

Ligand-based virtual screening technique employs the therapeutic information present in a known active compound rather than the structure of a target protein for the identification and optimization of lead. To find potentially active drug-like molecules for experimental evaluation, large biodata sets are investigated. Quantitative structure–activity relationships (QSAR), pharmacophore mapping, similarity substructure searching, and machine learning are examples of methods used in LBVS. Ligand-based visual screening of dual PMIX and PMX inhibitor 49c led to the identification of three leads from the ZINC database, namely; ZINC03351002, ZINC03417506, and ZINC02619047 [19]. Molecular dynamic simulation and post-analysis studies established these identified zinc compounds impose structural stability and compact architecture of the target enzyme PM IX, prohibiting the typical twist motion of the protease and keeping the ligand close to the catalytic aspartic dyad [19].

### 2.6. Pharmacokinetic Assessment of Antimalarial Compounds

The drug-likeness assessment of potential pharmaceutical compounds is an important stage in the drug discovery process. It evaluates the administration, distribution, metabolism, excretion, and toxicity of the compound of interest. The Lipinski rule of five is an online tool to predict the drug-likeness of a compound with parameters such as molecular weight, blood-brain permeability, gastrointestinal absorption, and lipophilicity, among others. This technique has been employed in previous studies [19,21] in the evaluation of leads.

### 2.7. Molecular Dynamic (MD) Simulation

This computational technique mimics the interaction of ligand–receptor complexes at the molecular level as though it is in a living system. MD simulations generate trajectories that are saved at 1 ps. Post-dynamic analysis on each of the systems under study is performed using the CPPTRAJ module within the AMBER 14/18 package. Subsequent graphical representations are conducted with the aid of the user interfaces of UCSF chimera and Microcal Origin analytical tools for the evaluation of structural dynamics such as the root mean square deviation (RMSD), root mean standard fluctuation (RMSF), and thermodynamic energy evaluation [26].

## 3. Aspartyl Acid Proteases (Plasmepsins) as Novel Drug Targets in *Plasmodium falciparum*

Recognition of plasmepsins in antimalarial drug discovery, though renowned, is yet to unknot an approved drug. Several proposed inhibitors were abandoned due to the limited potential of their therapeutic pathway. PMs are characterized by “two” aspartic acid residues in the active site. Presently there are ten classes of plasmepsins; PM I-X [27]. Plasmepsins are further categorized based on their preferred site of activity, inside or outside the host cell’s acidic food vacuole. Regardless, they collectively play a critical role in the survival and perpetuation of the infection [28] therefore, obstructing these processes could further expose the parasite to its vulnerable state, accentuating these enzymes as promising drug targets [29]. The enzymatic activities of the individual plasmepsins and their importance in drug discovery are expounded in Section 3.1 and Section 3.

**Figure 5:**
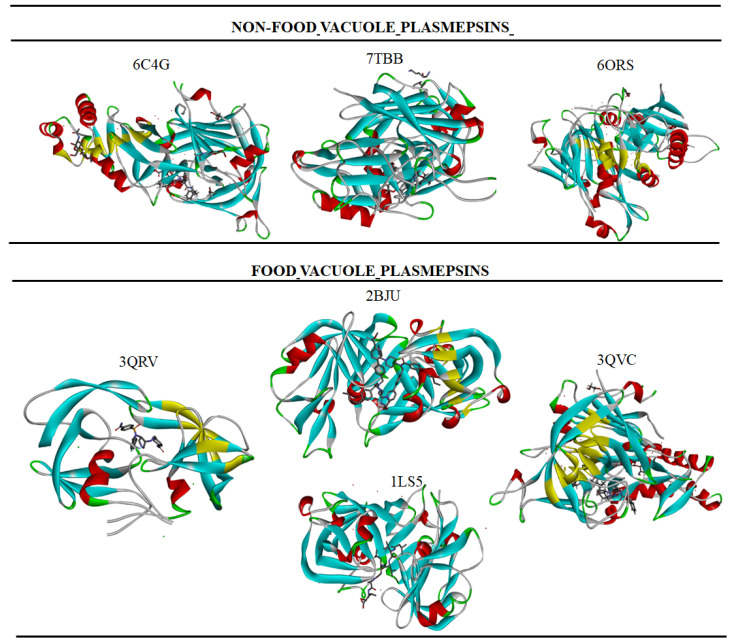
Crystallized structures and PDB code of complexes of *P. falciparum* and *P. vivax* Plasmepsins retrieved from the protein data bank (PDB) [35].

Hexapeptide statin-containing amino acid *Pepstatin A* showed aspartic protease inhibitory prowess, specifically against PMII. However, the adverse side effect upon the administration of *Pepstatin A* renders its clinical usage questionable. Small molecule competitive inhibitors 49c and WM382 have experimentally proven to exert dual inhibitory prowess on both PMIX and PMX [30]; the enzyme inhibitory mechanism underlying their inhibitory is unilateral, as both compounds cause rigidity of the proteases distorting the characteristic twist notion typical of all plasmepsins. X-ray crystalized structures of food vacuole and non-food vacuole plasmepsins with their corresponding codes at the protein data bank (PDB) are represented in Figure 5.

### 3.1. Food Vacuole Plasmepsin

*P. falciparum* is the only species that has numerous plasmepsins active within the food vacuole. The development of the intraerythrocytic stages of malarial parasites depends on hemoglobin (Hb) breakdown. Four aspartic proteases, PMI, PMII, Histo-aspartic acid (PMIII), and PMIV, are known to be involved in this process, which takes place inside an acidic digestive vacuole (DV) [31]. These enzymes have received considerable attention as potential antimalarial drug targets [32].

Hemoglobin degradation involves multiple stages whose initiation is specifically conducted by PMI and PMII. These two proteases cleave the native hemoglobin tetramer 54 between αPhe33-αLeu34 in the highly conserved hinge region, unraveling and exposing the protein to further degradation. This collective effort may enable efficient degradation of the molecule. Plasmepsin III, also known as HAP, is an active protease with a catalytic mechanism activity that mimics that of the aspartic acid proteases in hemoglobin degradation and seemingly has a high affinity for *Pepstatin A*, with a putative catalytic dyad comprising histidine and aspartic acid [33]. PMIV is involved in cytoskeletal protein processing and host cell remodeling. The food vacuole plasmepsin shows high levels of sequence similarities. The predicted coding sequences are 50–70% identical to each other at the amino acid level. High levels of sequence identity in both pro and mature areas imply that the emergence of PM I, II, and IV and HAP was caused by very recent gene duplication events [34]. Due to the functional redundancy within the FV plasmepsins, the use of selective inhibitors targeting a single plasmepsin will not effectively or efficiently kill the parasite.

### 3.2. Non-Food Vacuole Plasmepsin

The remaining members of the plasmepsin family PMV-PMIX are known as non-food vacuole plasmepsin as they are effective outside the acid food vacuole. Unlike the FV plasmepsins, this class of plasmepsins shows relatively lower structural similarity. Sequence alignment analysis of PMIX and PMX showed a structural similarity of 44.31% (unpublished paper). In comparison, PMVI-VIII are expressed in the vector during the parasite’s intraerythrocytic stages of sporozoite formation and motility. They, however, do not play a direct role in the transmission of the parasite within the human host [1]. PMVI-VIII are expressed in the vector during the parasite’s intra-erythrocytic stages of motility, formation of sporozoites, and midgut transversal and hence are not directly involved in the transmission of the malarial parasite within the human host. PMIX and PMX, on the other hand, are highlighted as mediators of hepatocellular egress, invasion, and transmission, making both proteases interesting targets for drug design. Enzymatic activities of plasmepsins in malaria pathogenesis are expressed in Table 3.

## 4. Advancement in the Search for Potent Plasmepsin Inhibitors

The food vacuole class of plasmepsins has gained much attention, yet there is no approved drug in this category [36]. Recent experimental investigation of PMIX and PMX, two important non-food vacuole plasmepsins, led to the discovery of 49c and WM382. Their essentiality in drug discovery is highlighted by their vital role, as stated in Table 2. Our previous studies focused on demonstrating the dual enzyme inhibitory prowess of WM382 on PMIX and PMX from a dynamic simulation perspective [20]. WM382 and its derivatives, ZINC03351002, ZINC03417506, and ZINC02619047, from virtual screening, impose structural compactness which stems from the mesh of hydrogen and hydrophobic interaction significantly with the catalytic dyad in the active pocket. From this study, we observed the catalytic dyad of PMIX (residues Asp32 and Asp281) contributed the highest binding energy toward the generated compounds. Their high affinity for the inhibitors ensured a stable conformation of the complexes for further molecular analysis.

Another headway inhibitor is compound 49c, similar to WM382, 49c causes structural compactness on both proteases, thereby prohibiting the characteristic twist motion of the flap residues [18,19]. Using computation techniques such as pharmacophore modeling and virtual screening, several leads with similar pharmacophoric moieties as these compounds have been generated from a database of drug-like compounds.

**Figure 6:**
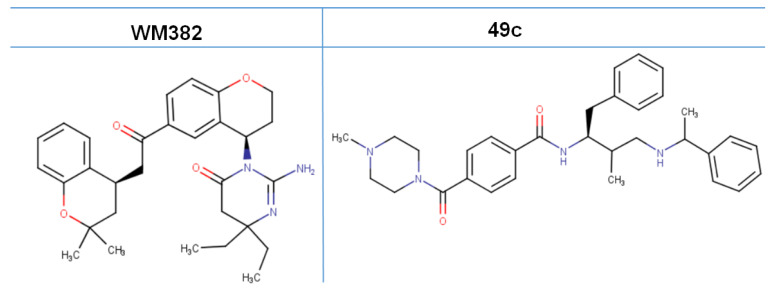
Chemical structure of non-food vacuole aspartyl acid protease inhibitors, WM382 and 49c [30].

The characteristic prowess of plasmepsin inhibitors to impose structural compactness on their target proteins restricts the typical twist motion, especially in the flap and hinge regions. The flap regions usually move away from the binding site exposing the catalytic dyad of the protease to the ligand. The chemical structures of compounds WM382 and 49c are represented in Figure 6.

### Limitations of Plasmepsin Inhibitors

The development of highly selective plasmepsin inhibitors remains a major obstacle in the drug discovery and development domain, as these proteases exhibit a high percentile of structural homogeneity. PMIX and PMX share over 40% structural similarity, including the catalytic dyad. Aside from the intra-family similarities, there is an alarming concern about the degree of the monotony of plasmepsins to human Cathepsin D, a pervasive lysosomal enzyme present in most cell types. The undesirable effect of *Pepstatin A*, a hexapeptide statin-containing amino acid, despite its inhibitory prowess against the food vacuole plasmepsins, was associated with off-targeting of the host Cathepsin D [37,38].

## 5. Conclusions

The lack of competitive inhibitors and the increasing challenge of parasite resistance to current antimalarial drugs has triggered the search for more intriguing drug targets and small molecule inhibitors to augment malaria chemotherapy. This current study discussed antimalarial drugs and transport proteins implicated in their resistance. Furthermore, we elaborated on the emergence of aspartic acid proteases as potential drug targets in the discovery and development of antimalarial drugs. In silico and bioinformatic techniques to speed up the antimalarial drug discovery and development to mitigate parasite resistance in malarial chemotherapy were as well expounded.

Hemoglobin degradation, hepatocellular egress, and dissemination of malaria parasites within the host are significant factors that influence the incision and virulence of malaria infection. Arresting the perpetrators of these acts is a step toward the relief and treatment of malaria. 49c and WM382, and experimentally proven small molecule inhibitors, are spearheading the course of plasmepsin inhibition. Both compounds have dual inhibitory activities against PMIX and PMX, the two vital non-food vacuole plasmepsins. 49c and WM382 form stable conformation within the active pocket of the proteases, which is initiated by hydrogen and hydrophobic interaction with the catalytic dyad and cause rigidity of the protease. Despite the advancement, the obstacle, however, is the structural homogeneity expressed among the family of plasmepsin as well as their similarity to human Cathepsin D, which hindered the clinical administration of *Pepstatin A.* To cover these challenges, computed-aided drug design (CADD) techniques have been incorporated. These in silico approaches aid in evaluating the pharmacokinetics and potency of drug candidates. Moreover, these methods predict the binding orientation and affinity of drug candidates to their respective targets, thereby reducing the incidents of off-targeting. Structure–activity relation and ligand/structure-based virtual screening augment the discovery of new drug candidates driven by the pharmacophoric moieties of known drugs or experimental inhibitors. Through molecular dynamic simulation, the enzyme inhibitory mechanism of potential drug candidates could be demonstrated and predict their usefulness in disease control.

## Figures and Tables

**Table 1:** Selected antimalarial drugs and their mode of action upon administration.

Antimalarial	Classification	Mode of Action
Artemisinin and derivatives; Dihydroartemisinin, Arteether, Artesunate, Artemether	Gametocytocidal	Involves the heme-mediated decomposition of the peroxide bridge to produce carbon-centered free radicals
Chloroquine	Blood schizonticidal	Chloroquine accumulates in the acidic food vacuole of intraerythrocytic trophozoites and thereby prevents hemoglobin degradation
Piperaquine	Blood schizonticidal	Accumulate in the parasite digestive vacuole and interfere with the detoxification of heme into hemozoin
Lumefantrine	Blood schizonticidal	Lumefantrine is believed to inhibit nucleic acid and the formation of beta-hematin by forming a complex with hemin
Curcumin	Blood schizonticidal	Curcumin inhibits the activity of enzyme and lipid peroxides

**Table 2:** List of proteins in *plasmodium* which has been implicated in the resistance of antimalarial chemotherapy.

Protein	Function	Location	Principal Drugs Affected
chloroquine resistance transporter (CRT)	Transporter	Membrane of food vacuole	Chloroquine Mefloquine, halofantrine, lumefantrine, artemisinin, quinine
Pgh1 (P-glycoprotein homologue 1) or MDR1 (multidrug resistance 1)	Transporter	Membrane of food vacuole	Mefloquine, halofantrine, lumefantrine, quinine
			Minor determinant
Dihydrofolate synthase (DHPS)	Folate pathway enzyme	Cytoplasm (principally)	Sulfadoxine, dapsone
Dihydrofolate reductase (DHFR)	Folate pathway enzyme	Cytoplasm (principally)	Pyrimethamine, proguanil, chlorproguanil
Cytochrome *b*	Subunit of complex III (cytochrome *bc*_1_ complex) electron transport chain	Mitochondrion	Atovaquone
ATP6 (sarco/endoplasmic reticulum calcium-dependent ATPase [SERCA] orthologue)	Membrane-bound Ca^2+^ -transporting ATPase	Membranous structures within cytoplasm	Artemisinin
Kelch13	Endocytosis pathway protein	Cytoplasm	Artemisinin

**Table 3:** Role of the classes of plasmepsins in malaria pathogenesis.

Class of Plasmepsin	Function
Food vacuole plasmepsin	**PMI** Hemoglobin digestion and degradation
	**PMII** Hemoglobin digestion and degradation. Cytoskeletal protein processing and host cell remodeling
	**Histo-aspartic protease (HAP)** An active protease with a novel catalytic mechanism in hemoglobin degradation
	**PMIV** Hemoglobin digestion and degradation. Cytoskeletal protein processing and host cell remodeling
Non-food vacuole plasmepsin	**PMV** Responsible for the export of effector proteins mediated to the host cell.
	**PMVI-VIII** Expressed in the vector during the parasite’s intra-erythrocytic stages of motility, formation of sporozoites, and midgut transversal and hence are not directly involved in the transmission of the malarial parasite within the human host
	**PMIX and PMIX** Egress, invasion, and spread of malaria parasite

## Data Availability

Not applicable.
